# Red Blood Cells in Normal and Pathological States: Redox Reactions of Hemoglobin

**DOI:** 10.3390/molecules31030444

**Published:** 2026-01-27

**Authors:** Krzysztof Gwozdzinski, Anna Pieniazek, Lukasz Gwozdzinski

**Affiliations:** 1 Department of Oncobiology and Epigenetics, Faculty of Biology and Environmental Protection, University of Lodz, 90-236 Lodz, Poland; krzysztof.gwozdzinski@biol.uni.lodz.pl (K.G.);; 2 Department of Pharmacology and Toxicology, Medical University of Lodz, 90-752 Lodz, Poland

**Keywords:** red blood cells, hemoglobin, oxidative damage, heme, inflammation, diabetes mellitus

## Abstract

Red blood cells (RBCs) play a key role in vascular origin pathologies such as nephropathy, retinopathy, and neuropathy. Altered RBCs also occur in the case of hereditary spherocytosis, hemoglobinopathies, sickle cell disease, thalassemia and hemolytic anemia. The consequence of damage to the cell membrane and cytoskeleton are changes in RBC deformability, which play an important role in microcirculation. In turn, oxidative changes in hemoglobin lead to impaired oxygen transport to cells and tissues and, consequently, to ischemia and hypoxia. In this review, we discuss the structure of normal and pathological RBCs, including, more broadly, red blood cells occurring in type 2 diabetes. We present factors that play a major role in RBC damage in this pathology. Finally, we characterize the participation of hemoglobin and heme in the induction of oxidative damage to biological material, including RBCs.

## 1. Introduction

Red blood cells (RBCs), transporting oxygen to cells and tissues, are associated with the reversible binding of oxygen to hemoglobin. In addition to the high partial pressure of oxygen in the lungs, RBCs are exposed to external and internal oxidizing factors. Oxidative damage leads to changes in its structure and function, which in turn causes disorders in oxygen delivery. The main endogenous source that supplies the superoxide anion radical (O_2_^•−^), a precursor of reactive oxygen species (ROS), is autoxidation of hemoglobin. In a healthy organism, about 3% of hemoglobin is converted to methemoglobin daily with the release of O_2_^•−^ [1]. Partially oxidized hemoglobin exhibits a distinct conformation that influences hemoglobin–membrane interactions, particularly involving the band 3 protein. In turn, autoxidation of partially oxidized hemoglobin bound to the membrane allows for easier release of ROS from red blood cells [2]. Additionally, RBCs can produce nitric oxide (^•^NO), with the participation of nitric oxide synthases NOS [3]. Nitric oxide, in reaction with the superoxide anion radical, generates peroxynitrite, a strong oxidizing and nitrating agent. Interestingly, ROS generation is strongly enhanced under hypoxic conditions [2]. In addition to the endogenous source, red blood cells can also be damaged by ROS originating from the endothelium and phagocytic cells. RBCs possess well-organized antioxidant systems that include both enzymes and low-molecular-weight antioxidants. Antioxidant enzymes consist of superoxide dismutase (CuZnSOD), catalase (CAT), glutathione peroxidase (GPx), peroxyredoxin 2 (Prx-2) and other enzymes, including glutathione reductase (GR) and methemoglobin reductase. Low-molecular-weight antioxidants include glutathione (GSH), ascorbate (ASC), α-tocopherol (α-TOH), β-carotene (β-Car), and others. Additionally, red blood cells utilize the pentose phosphate pathway, which provides a crucial reducing agent, NADPH, that supports antioxidant systems. Pathologies also affect the production of ROS in red blood cells; currently, over 1000 disorders of hemoglobin synthesis and/or structure are known [4]. The most well-known of these are sickle cell disease, thalassemia, hemoglobinopathies, favism, and blood concentrates intended for transfusion, which are characterized by a reduced antioxidant level.

This review discusses the structure of normal and pathological red blood cells, including, more broadly, red blood cells occurring in type 2 diabetes. Factors that play a major role in RBC damage in this pathology are presented. Finally, the participation of hemoglobin and heme in the induction of oxidative damage to biological material, including RBC, is characterized.

### 1.1. Red Blood Cells

RBCs are among the most abundant cells in the blood, their lifespan in the bloodstream is 120 days. Old blood cells are captured in the spleen, where they are decomposed by macrophages. However, some of these cells may undergo intravascular lysis, which results in the release of hemoglobin. Hemoglobin is readily autoxidized and subsequently decomposed into heme. To prevent the toxic effects of hemoglobin and heme released from RBCs, these substances are bound by acute phase proteins such as haptoglobin and hemopexin. However, the binding capacity of both forms is limited by the capacity of acute-phase proteins, exceeding which leads to the accumulation of various forms of Hb and heme. Intravascular lysis of red blood cells typically occurs in various pathologies. To maintain normal blood flow, RBCs undergo deformation (deformability). RBC deformability is determined by the characteristic biconcave shape, but is also related to the structure of the plasma membrane, the properties of the membrane cytoskeleton, and the viscosity of the cytosol. Pathological conditions can significantly affect the changes related to the rheology of red blood cells. In addition to lipids, the bilayer also includes integral transmembrane proteins, which are associated with the cytoskeleton through linker proteins [5]. An important factor influencing plasma membranes is the fluidity of the plasma membrane, which is related to the presence of cholesterol, but also to unsaturated fatty acids that are part of the lipids. Fluidity is the physical state of the membrane, which is related to the degree of lipid packing in the membrane, the presence of proteins and cholesterol, and protein-lipid interactions in the membrane. It has been shown that the molecular structure of the lipid bilayer of the red blood cell membrane is altered in patients with diabetes [6]. The lipid bilayer maintains phospholipid asymmetry regulated by proteins such as scramblases and flippases [7]. In turn, integral transmembrane proteins that pass through both lipid monolayers act as transport proteins, receptors, signaling molecules, and carriers of red blood cell antigens. Among the most abundant transmembrane proteins are band3 and gycophorin. Band 3 connects the outer surface of the membrane with the cytoskeleton, is a transport protein for anion exchange in RBCs, and also participates in the transport of carbon dioxide [5]. Disturbances in the structure of proteins involved in the stability of the RBC membrane structure lead to reduced deformability and a shortened lifespan of red blood cells [8]. In addition to oxygen transport, RBCs participate in hemostasis and thrombosis in physiological and pathophysiological conditions [9,10]. Prothrombotic properties of RBCs are associated with various factors, such as the release of procoagulant substances, e.g., ADP and extracellular vesicles with exposed phosphatidylserine (PS), initiation of platelet aggregation, participation in vascular clot, and impairment of fibrinolysis [11,12,13]. Both quantitative and qualitative anomalies in red blood cells contribute to venous thrombosis and arterial thrombosis. In both cases of thrombosis, an important factor is blood viscosity, as well as increased adhesion to the vessel or artery wall, margination, activation, and aggregation of platelets. RBCs promote platelet margination, which leads to increased platelet-vessel interactions, and RBCs enhance platelet activation and aggregation [14]. In the case of venous thrombosis, RBC aggregation (rouleaux) occurs at low shear rates, which increases blood viscosity [14]. Interestingly, both low and high hematocrit are U-shaped risk factors for stroke/transient ischemic attack in older women [14]. In turn, increased RBC membrane stiffness or aggregation can cause damage to the vascular endothelium, leading to thrombosis, which occurs in sickle cell disease, thalassemia, hemolytic anemia, malaria, and in sepsis or COVID-19 [9,14,15].

### 1.2. Red Blood Cells in Pathologies

The most important pathologies associated with abnormal red blood cell structure and function include: anemia, low level/activity of enzymes in RBCs, e.g., G6PD, disorders in the structure and function of the RBCs plasma membrane, e.g., hereditary spherocytosis, hemoglobinopathies, e.g., sickle cell disease and thalassemia, hemolytic anemia, anemias caused by nutrient deficiency, e.g., anemia caused by low iron and folic acid levels, abnormal heme production, e.g., sideroblastic anemia, hemochromatosis (accumulation of iron in the body), as well as polycythemia (excess RBCs). In addition to pathology, usually hereditary, structurally altered blood cells are also found in chronic kidney disease (CKD) and diabetes mellitus (DM), although diabetes often accompanies CKD [16,17]. For example, the lifespan of red blood cells is significantly shorter in CKD and DM compared to the healthy group and depends on the disease state [18,19]. The altered structure of the RBC plasma membrane is found, among others, in hemolytic anemias, such as hereditary spherocytosis (HS), hereditary elliptocytosis (HE), hereditary pyropoikilocytosis (HPP), and Southeast Asian ovalocytosis (SAO) [20]. In turn, in the case of sickle cell disease, disturbances in the structure of hemoglobin lead to its polymerization, which causes changes in membrane deformability and increased adhesive properties of RBCs [21]. Disturbed adhesion leads to the occlusion in capillaries, which causes disease exacerbation and organ damage. Hereditary spherocytosis (HS) involves mutations in genes that encode transmembrane or cytoskeletal proteins, such as spectrin, ankyrin, protein 4.2, and band 3. The consequences of this are a decrease in the membrane surface, an increase in the stiffness of the plasma membrane, and disturbances in the mechanical stability of the membrane [8]. Changes in the structure of RBCs can lead to hemolysis in the circulation and/or disturb blood rheology. Abnormal RBCs can cause anemia with reticulocytosis, endotheliitis, and microvascular obstruction.

### 1.3. Red Blood Cells in Diabetes Mellitus

The development of type 2 diabetes leads to a significantly increased risk of atherosclerosis and cardiovascular diseases. With the progression of diabetes, the number of normal biconcave erythrocytes decreases, and the number of deformed erythrocytes increases. The change in the ratio of normal to abnormal RBCs decreases, which leads to an increased risk of diabetic complications [22]. Hyperglycemia leads to the glycosylation of RBC membranes, resulting in their stiffening and a reduction in RBC deformability. [23]. Impaired RBC deformability led to microcirculation disorders [24]. Furthermore, a reduction in the negative surface charge of cells resulted in increased RBC aggregation [25]. The buildup of cells can disrupt blood flow, resulting in inadequate tissue perfusion and oxygen transport. This condition causes local tissue ischemia and hypoxia. Reduced membrane fluidity resulting from increased non-enzymatic glycation and lipid peroxidation caused by reactive oxygen species may be an indicator of type 1 diabetic retinopathy [26]. The change in the fluidity of diabetic erythrocytes increases their aggregation and weakens their deformability, which leads to metabolic disorders. Therefore, increased aggregation and reduced deformability and fluidity of erythrocytes caused by hyperglycemia can lead to high blood viscosity and coagulation, which results in microcirculation disorders and becomes an important cause of macro- and microvascular complications of diabetes [27]. In diabetes, hemoglobin undergoes glycation to form glycated Hb (HbA1c), which is used as an important diagnostic indicator of diabetes. HbA1c has a higher affinity for O_2_ than normal Hb, but reduced oxygen release into cells and tissues (limited oxygen transport by RBCs) [28,29,30]. A positive correlation between HbA1c concentration and diabetic retinopathy (DR) has been reported, and local hypoxia promoted an increase in the thickness of the glomerular basement membrane, initiating DM [31]. It has also been reported that high HbA1c concentrations are associated with both macrovascular and microvascular diseases [32]. Hyperglycemia leads to vascular and multi-organ complications, which are caused by excessive ROS production [33,34,35,36]. The main source of ROS in diabetes is mitochondria (Mtch), because high glucose concentration increases the metabolic input to Mtch, overwhelming the electron transport chain (ETC), which causes mitochondrial hyperpolarization and overproduction of ROS [33,37]. To achieve ROS overproduction at high glucose concentration, an increase in intracellular calcium ion concentration is necessary [38]. Red blood cells are sensitive to ROS, which causes oxidation of proteins and lipids. Cytoskeletal proteins and other membrane proteins are particularly sensitive to ROS. ROS leads to changes in RBC structure and function [39]. In hyperglycemia, patients have an increased ROS, while the antioxidant capacity of RBCs is reduced [40]. Overproduction of ROS is accompanied by a decrease in glutathione (GSH) levels, which in patients with dyslipidemia is 30% lower than in healthy individuals [41]. In addition, there is also a decrease in antioxidant capacity through a decrease in the activity of enzymes such as superoxide dismutase, catalase, glutathione peroxidase, glutathione reductase, glutathione levels, vitamins, lipid peroxidation, nitrite concentration, non-enzymatic glycosylated proteins and hyperglycemia in diabetes [40,42]. In addition to the decrease in GSH levels, a decrease in low-molecular-weight antioxidants is also observed, which includes vitamin E, GSH, and ascorbic acid, while the average level of lipid peroxidation was doubled [41]. All these factors result in damage to RBCs due to oxidative stress (OS), which is closely linked to the microvascular complications of diabetes [43]. In DM, changes in RBC structure, changes in the redox state, and increased oxidative changes are observed. In vitro glycated RBCs were characterized by reduced antioxidant defense, including the activity of antioxidant enzymes. Increased phagocytosis of RBCs by endothelial cells was also observed, which was associated with an increased level of phosphatidylserine exposed in the outer monolayer of the membrane, leading to eryptosis [44]. The oxidative damage observed in vitro in glycated RBCs was similar to RBCs isolated from diabetics [45]. Therefore, glycation, oxidative stress, and uremic toxins significantly contribute to RBC dysfunction, leading to a shortened life span in the circulation, anemia, and the development of various complications associated with DM.

It is known that chronic hyperglycemia in diabetes (DM) can lead to numerous complications related to microvascular disorders, leading to nephropathy, retinopathy, and neuropathy [46]. In addition, diabetes often involves changes in various hematological parameters determining the structure, function, and metabolism of blood cells, such as red blood cells, white blood cells (WBCs), and platelets [47,48]. Increasing evidence indicates the involvement of red blood cells in disorders related to DM-related complications. In diabetes, morphological, enzymatic, and biophysical changes in RBCs are observed, which lead to their early removal from the circulation. The changes induced in RBC structure are caused by oxidative stress, uremic toxins, and carbamylation, which underlie the shortened survival of RBCs in hyperglycemia [17]. Additionally, these factors can affect RBC metabolism, procoagulant RBC phenotype, RBC-induced endothelial cell dysfunction, and changes in RBC deformability and aggregation, as well as red blood cell death signaling. Furthermore, changes in the concentration of fatty acids in the RBC affect the fluidity of the cell membrane, but also its deformability. In diabetes, significant disturbances in lipid composition are observed, which are characterized by higher levels of saturated fatty acids, cholesterol, sphingolipids, and sphingomyelin. The cholesterol to phospholipid ratio is also higher. These changes lead to increased stiffness of RBC plasma membranes [49]. In diabetes, redox balance disorders, disruptions in the normal structure of the RBC plasma membrane, and changes in the expression of membrane transporters, including the activity of the sodium-potassium pump, calcium ATPase, and acetylcholinesterase, are observed [41]. RBC dysfunction may play an important role in microangiopathy in DM [17]. Diabetes promotes the development of vascular calcification (VC). Furthermore, in central vascular disease in patients with chronic kidney disease (CKD), it may be associated with VC. It has been shown that VC is initiated by the stimulation of proinflammatory factors, which consequently disrupts endothelial function and triggers similar mechanisms in the development of both diseases [50]. Moreover, increased levels of ammonia, citrulline, urea, uric acid, and ornithine were found in RBCs in diabetic patients, while the arginine level was significantly lower than in healthy individuals [51].

### 1.4. Effects of Methylglyoxal as a Uremic Toxin

Methylglyoxal (MG) as a uremic toxin and a product of lipids, proteins and glucose metabolism. Its intracellular concentration is 1–4 μM. As an aldehyde, it reacts with thiol and amino groups to form covalent bonds with proteins and nucleic acids, DNA, and RNA, forming advanced glycation end products (MG-AGE). In addition to diabetes, MG and MG-AGE are associated with the onset and progression of many pathologies, such as liver and kidney diseases and cancer [52]. Methylglyoxal in diabetes leads to cell dysfunction [53]. In diabetes mellitus, elevated MG levels may occur in RBCs and plasma [53]. MG disturbed the energy and oxidative balance and initiated changes in RBC deformability and elongation [54]. In the case of developing DM nephropathy, in addition to elevated MG levels, not only are elevated levels of extracellular phosphates observed, but also the levels of other uremic toxins, such as p-cresol, indoxyl sulfate, and acrolein, which initiate RBC damage, leading to death [55,56,57,58]. These uremic toxins may play a role in the development of diabetic nephropathy and other complications of diabetes [59]. MG initiated increased membrane sensitivity, hemolysis, and a decrease in amino groups in RBCs. In the case of leukocytes, it caused DNA damage, decreased cell viability, and increased levels of glycated products. In platelets, MG inhibited the activity of NTPDase, 5’-nucleotidase, and adenosine deaminase (ADA) enzymes without affecting the levels of free amino groups [60]. These results indicate that MG damages different blood cells through different mechanisms of action. It has been shown that in DM, there is a high percentage of RBCs exposing phosphatidylserine in the outer lipid monolayer of the cell membrane. Such cells are recognized, taken up from the circulation, and degraded by macrophages. However, red blood cells exposing PS can also be bound to the blood vessel wall, disturbing blood flow in the microcirculation [61,62]. It was shown that treatment of RBCs with methylglyoxal significantly increased the percentage of RBCs exposing PS at the concentration (0.3 μM) found in DM patients. Furthermore, methylglyoxal inhibited glycolysis, lowering ATP and GSH concentrations in RBCs, and impaired energy production and antioxidant defense [54]. Uremic toxins, together with chronic inflammation, oxidative stress, damaged RBCs, and endothelial cells (ECs) dysfunction, as well as blood hemodynamic changes, which are associated with changes in hematological parameters, lead to the pathophysiology of cardiovascular and renal function [27].

### 1.5. Prooxidant Action of Hemoglobin

Hemoglobin (HbO_2_, Hb(Fe^2+^), oxyHb) is a respiratory protein with a molecular weight of 64,000 to 65,000, found primarily in red blood cells. Its main function in the body is to transport oxygen to cells and tissues. Hemoglobin is a tetramer composed of two alpha and two beta globin chains. Each globin chain contains a heme group, which contains an iron(II) ion that reversibly binds an oxygen molecule. During RBC lysis, hemoglobin is released into the external environment, the plasma. The released HbO_2_ is much more sensitive to oxidation, which leads to methemoglobin (MetHb) and the reactive radical (cation radical) ferryl form (Hb(Fe^4+^=O^•+^). In turn, hemoglobin oxidizes to the ferryl form (Hb(Fe^4+^=O). Both ferryl forms can lead to inflammation and oxidation (peroxidation) of lipids, proteins, and other macromolecules.

The consequence of the autoxidation of hemoglobin is production of superoxide anion radical, which undergoes spontaneous or catalyzed dismutation to hydrogen peroxide (H_2_O_2_) [1].

Hb(Fe^2+^)O_2_ → Hb(Fe^3+^)+ O_2_^•−^

The resulting hydrogen peroxide, if not removed by catalase, glutathione peroxidase or/and peroxiredoxin-2, can oxidize hemoglobin or methemoglobin [MetHb, Hb(Fe^3+^)] to the ferryl form (HbFe^4+^=O) and the corresponding radical oxyferryl form (^•^HbFe^4+^=O), which are strong oxidants.

Hb(Fe^2+^) + H_2_O_2_ → Hb(Fe^4+^=O) Hb(Fe^3+^) + H_2_O_2_ → Hb(Fe^4+^=O)^•+^

The lifetime of the ferryl radical form is short, expressed in milliseconds, while the ferryl form is present for minutes or even hours. The cation radical form undergoes rapid deprotonation, creating the radical ^•^R(Fe^4+^=O) [63]. This form was identified in the blood of healthy volunteers using the EPR spectroscopy method [64]. The oxoferryl form has pseudocatalase properties that break down hydrogen peroxide.

Hb(Fe^4+^=O) + H_2_O_2_ → Hb(Fe^3+^) + O_2_^•−^

All forms containing iron ions in higher oxidation states are easily decomposed [65]. The ferryl form of hemoglobin reacts further with H_2_O_2_, forming heme degradation products and free iron. The ferryl form of Hb, as a result of intramolecular electron transfer between Fe^4+^=O ion and amino acid residues in globin, can form globin radicals. The ferryl radical form can undergo additional oxidative reactions. The radical globin reaction results in cross-linking of oxidized hemoglobin derivatives [66].

Hb(Fe^4+^=O) + 2H^+^ → (HbFe^3+^)^•+^ + H_2_O

(HbFe^3+^)^•+^ + (HbFe^3+^)^•+^ → (HbFe^3+^)^+^ − (HbFe^3+^)^+^

Nitric oxide (^•^NO) is produced by numerous sources, such as dietary nitrates and nitrites and by oxidation of L-arginine in the presence of NOS. ^•^NO has a strong affinity for iron (II) ions, including deoxyhemoglobin.

Hb + ^•^NO → HbNO

However, in the presence of oxygen, nitrosohemoglobin (HbNO) is oxidized to methemoglobin, similarly to the reaction of oxyhemoglobin with ^•^NO.

HbFe^2+^O_2_ + ^•^NO → HbFe^3+^OONO → HbFe^3+^ + NO_3_^−^

During this reaction, the peroxynitrito-complex HbFe^3+^OONO is formed indirectly, which is decomposed to methemoglobin and nitrate ion. The reaction rate constant is high and equal to k = 8.9 × 10^7^ M^−1^s^−1^ [67]. Nitric oxide also reacts with methemoglobin with a rate constant k = 1.71 × 10^3^ M^−1^ s^−1^ to form the HbFe^2+^(NO^+^) complex [68].

HbFe^3+^ + ^•^NO → HbFe^3+^NO → HbFe^2+^(NO^+^)

Nitric oxide can also inactivate the ferryl form of hemoglobin. The reaction rate constant is high and equal to k = 2.4 ± 0.2 × 10^7^ M^−1^ s^−1^ [68].

Hb (Fe^4+^=O) + ^•^NO → HbFe^3+^ONO → HbFe^3+^ + NO_2_^−^

**Figure 1:**
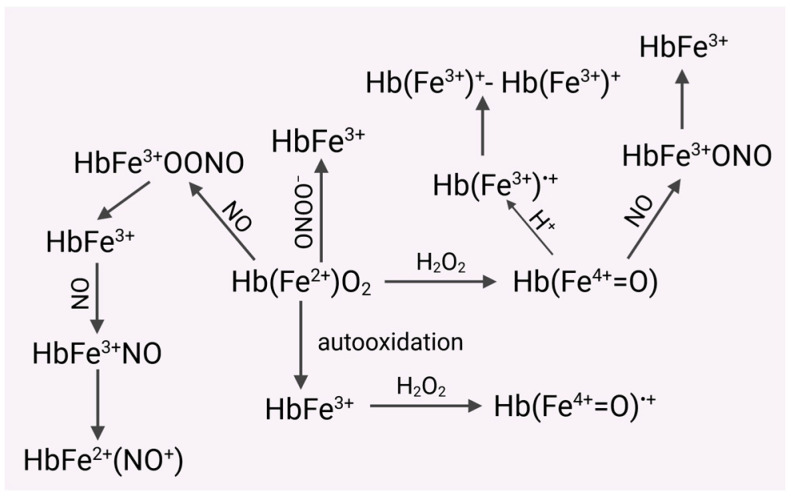
Hemoglobin released from RBCs is much more susceptible to oxidation to form methemoglobin (MetHb). Both HbO_2_ and Met Hb are oxidized by hydrogen peroxide, respectively, to the ferryl form of Hb ((Hb(Fe^4+^=O) and the radical ferryl form (Hb(Fe^4+^=O^•+^). HbO_2_ can be oxidized by nitric oxide (NO) to MetHb and then to the complex HbFe^2+^(NO^+^). In turn, the ferryl form of Hb can form a cation radical with an iron ion in the third oxidation state. Recombination of both radicals leads to the formation of dimers of hemoglobin derivatives (HbFe^3+^)^+^-(HbFe^3+^)^+^. HbO_2_ also reacts with peroxynitrite to form MetHb. Nitric oxide eliminates the toxic ferryl form of Hb, converting it into MetHb.

Importantly, the rate constants of hemoglobin with nitric oxide in RBCs can be about 3000 times lower compared to extracellular hemoglobin [69]. It has been shown that under certain conditions (expression of ^•^NO), irreversible consumption of ^•^NO by hemoproteins may play a protective role against its harmful effects (Figure 1) [70,71,72,73].

In addition to the reaction with heme iron in hemoglobin, nitric oxide can be bound by the cysteine residue Cysβ93, which occurs in globin chains. This reaction produces S-nitrosohemoglobin (HbSNO) [74,75]. RBCs participate in vasodilation, which is associated with autoregulation of blood flow and consists of vasodilation, leading to increased oxygen delivery in hypoxia. This mechanism involves the binding of nitric oxide to hemoglobin to form nitrosohemoglobin. The formation of HbSNO is particularly important in the regulation of local blood flow, because the release of nitric oxide from S-nitrosohemoglobins is coordinated with the release of oxygen [76]. It has been shown that RBCs not only bind to NO, but also transport and release NO in the circulatory system. Therefore, hemoglobin not only transports oxygen but also affects vasodilation. During the deoxygenation of hemoglobin, NO is also released, which regulates blood flow in the vessels [77]. Although the synthesis of nitric oxide is usually associated with vascular endothelial cells, it has been shown that red blood cells have NOS (eNOS), which occurs in the cell membrane and in the cytoplasm. The activity of this enzyme is regulated by L-arginine, calcium, and also by phosphorylation with the participation of PI3 kinase. NOS activity is associated with RBCs membrane deformability and inhibition of platelet activation. The activity of this enzyme in RBCs is comparable to the activity in cultured human endothelial cells [3]. Human red blood cells also have functional arginase and its inhibition increases the release of nitric oxide with the participation of eNOS in RBCs. The functional action of arginase was studied in an ex vivo model of myocardial ischemia–reperfusion injury. Arginase inhibitors administered in blood or plasma from RBCs were shown to significantly improve functional recovery after ischemia in rat hearts. However, this effect was not observed when arginase 1 inhibitors were administered after ischemia in buffer solution or plasma. Interestingly, the protective effect of arginase inhibition was lost in the presence of a NOS inhibitor. These results confirmed that RBCs contain functional eNOS and exhibit eNOS-like activity in NO release. Furthermore, it has been shown that this process is tightly controlled by arginase 1, which plays an important role during ischemia–reperfusion [78]. However, overproduction of nitric oxide can be harmful, as nitric oxide is easily oxidized to ^•^NO_2_ (reaction rate constant in aqueous solution at 37 °C k = (2.1 ± 0.3) × 10^6^ M^−2^ s^−1^) [79]. Nitrogen dioxide (^•^NO_2_) is highly toxic and is also a strong oxidant and nitrating agent [80]. As an oxidant, ^•^NO_2_ can initiate the process of lipid peroxidation, and as a nitrating agent, it leads to the formation of 3-nitrotyrosine residues in peptides and proteins. The reaction of ^•^NO with ^•^NO_2_ produces nitrous acid anhydride (N_2_O_3_), which reacts with water to form nitrous acid, and with thiols to form nitrosothiols RSNO [81]. In addition, the reaction with amines leads to nitrosamines. The nitrosates thiols, and amines reaction involves nitrosonium cation NO^+^.

Another agent with strong oxidizing and nitrating properties is peroxynitrite ion (ONOO^−^) and/or peroxynitrous acid (ONOOH, pKa = 6.8), which is formed in the diffusion-controlled reaction of nitric oxide with superoxide anion. reaction rate constant (k = (4–16) × 10^9^ M^−1^ s^−1^) is about 3 times higher than the rate constant of dismutation of superoxide anion radical catalyzed by SOD.

^•^NO + O_2_^•−^ → ONOO^−^

Peroxynitrite is a strong oxidizing and nitrating agent, which is associated with the decomposition of peroxynitrous acid (ONOOH, pKa = 6.8). Decomposition of peroxynitrous acid leads to a complex (30%) with strong oxidizing-nitrating properties containing hydroxyl radical (HO^•^) and nitrogen dioxide (caged radical pair) and nitric acid [82,83].

ONOOH → [HO^•^ -----^•^NO_2_] + NO_3_^−^

Peroxynitrite is the main target of ONOO- initiates lipid and protein peroxidation, and protein nitration, and also reacts with thiols such as glutathione and cysteine. Thiols are the main scavengers of peroxynitrite. It nitrates tyrosine and tryptophan residues in peptides and proteins and guanine residues in DNA, as well as aliphatic fatty acids and sugars. Since the concentration of glutathione (5–10 mM) in cells is much higher than that of cysteine, it is the main scavenger of peroxynitrite. ONOO^−^ also shows high affinity for selenoproteins, e.g., glutathione peroxidase (GPx), and also for iron-sulfur complexes such as 4Fe-4S clusters. Such a cluster occurs, for example, in aconitase, and its reaction with ONOO^−^ leads to the inactivation of the enzyme. Peroxynitrite also reacts with the active site of alcohol dehydrogenase, which contains the zinc–sulfur complex. Moreover, ONOO^−^ reacts with heme in hemoglobin and with cytochrome c and peroxidases with reaction rate constants of 10^4^–10^6^ M^−1^ s^−1^ [82].

Peroxynitrite formed intravascularly can diffuse through RBCs plasma membranes and react with oxyhemoglobin at a rate constant (2 × 10^4^ M^−1^ s^−1^) [84]. In the case of an excess of HbO_2_ relative to ONOO^−^, one-electron oxidation of HbO_2_ to MetHb occurs with the formation of nitrate, superoxide radical, and hydrogen peroxide [85].

2HbFe^2+^O_2_ + ONOO^−^ + 2H^+^ → 2HbFe^3+^ + NO_3_^−^ + O_2_^•−^ + H_2_O_2_

It has also been shown that peroxynitrite can also form ferryl forms of hemoglobin with an efficiency of about 10%, which can decompose to MetHb. Spin trapping studies using two spin traps, 2-methyl-2-nitrosopropane (MNP) and 5,5-dimethyl-1-pyrroline-N-oxide (DMPO) showed the formation of paramagnetic adducts of tyrosyl and cysteinyl radicals. DMPO inhibited the formation of dimerization products, which resulted in the formation of DMPO-hemoglobin adducts. Interestingly, nitration of hemoglobin was not observed. However, in the case of an excess of peroxynitrite in HbO_2_, nitrohemoglobin was formed. The authors suggest that oxyhemoglobin may play a role as an intravascular absorber of peroxynitrite [85]. In the presence of physiological concentration of CO_2_ (1.2 mM), peroxynitrite forms CO_2_/ONOO complex [^•^NO_2_----^•^OCO_2_^−^] a strongly oxidizing adduct (second-order rate constant in the order of 10^5^ M^−1^ s^−1^), which decomposes to form nitrogen dioxide and carbonate anion radical (cage radical pair) [83].

ONOO^−^ + CO_2_ → [^•^NO_2_------^•^OCO_2_^−^] → ^•^NO_2_ + ^•^OCO_2_^−^

The presence of carbon dioxide affects the reactivity of peroxynitrite. Of course, the carbonate radical anion has much lower reactivity than the hydroxyl radical. However, the presence of CO_2_ influences the greater nitrating properties of peroxynitrite [83]. It has been shown that not the adduct CO_2_/ONOO^−^, but its decomposition products, i.e., ^•^NO_2_ and ^•^OCO_2_^−^, react with HbO_2_ [86]. Recently, the possibility of creating methemoglobin from the reaction of oxyhemoglobin with ^•^NO was used as a contrast agent in magnetic resonance angiography (MR) in the assessment of vascular pathology. Methemoglobin causes an increase in the signal intensity of the T1-weighted blood MR image and may be a safe and effective alternative to toxic gadolinium-based contrast agents [69]. Gadolinium compounds accumulate in the brain and other organs of healthy individuals for a long time. MetHb remained in the bloodstream for at least 90 minutes after the source of ^•^NO was removed. Then, natural conversion to hemoglobin occurred by NADPH-dependent methemoglobin reductase. The source of ^•^NO can be sodium nitrite, or a blood sample can be exposed extracorporeally using high concentrations of ^•^NO and then reintroduced rich in MetHb into the patient’s bloodstream [69]. The idea of using MetHb as a contrast agent is interesting. However, nitrogen dioxide is formed during the reaction of HbO_2_ with nitrites [87].

HbO_2_ + NO_2_^−^ + 2H^+^ → HbFe^3+^ + ^•^NO_2_ + H_2_O_2_

In this reaction, an intermediate compound is initially formed, which is a complex of MetHb and hydrogen peroxide, HbFe^3+^HOOH, considered equivalent to the perferryl radical [88].

HbO_2_ + NO_2_^−^ → HbFe^3+^HOOH + ^•^NO_2_

This complex reacts with another nitrite ion to form another ^•^NO_2_ molecule [88].

HbFe^3+^HOOH + NO_2_^−^ → HbFe^4+^O + ^•^NO_2_ +H_2_O

However, a reaction is also postulated in which nitrogen dioxide is not formed but only hydrogen peroxide [89].

2HbO_2_ + 2NO_2_^−^ + 2H^+^ → 2HbFe^3+^ + H_2_O_2_ + 2NO_3_^−^

The resulting hydrogen peroxide oxidizes MetHb to the ferryl radical cation [Hb(Fe^4+^=O)^•+^], which then oxidizes the nitrite ion to nitrogen dioxide [87,89].

Hb(Fe^4+^=O)^•+^ + NO_2_^−^ + 2H^+^ → HbFe^3+^ + ^•^NO_2_ + H_2_O

Nitrogen dioxide oxidizes the hemoglobin molecule to the ferryl radical form, but a short-lived peroxynitrate adduct is formed transiently [90].

HbO_2_ + ^•^NO_2_ → ^•^HbFe^4+^OONO_2_

^•^HbFe^4+^OONO_2_ → ^•^HbFe^4+^O + NO_3_^−^

The rate constant of this reaction was 6.9 × 10^5^ M^−1^ s^−1^ [89] and was two orders of magnitude lower compared to myoglobin (4.5 ± 0.3) × 10^7^ M^−1^ s^−1^ [90].

A single-electron oxidation reaction of hemoglobin by ^•^NO_2_, in which molecular oxygen is released, is also possible [87].

HbO_2_ + ^•^NO_2_ → HbFe^3+^ + O_2_ + NO_2_^−^

Nitrogen dioxide in water undergoes a disproportionation reaction to form nitrite and nitrate ions.

^•^NO_2_ + H_2_O → NO_2_^−^ + NO_3_^−^ +2H^+^

We have shown that nitric oxide causes oxidative damage to red blood cells. NO initiated a significant increase in membrane fluidity at different depths of the outer lipid monolayer. In addition, significant changes were observed in the conformational state of cytoskeletal proteins, the spectrin-actin complex. These changes were accompanied by an increase in lipid peroxidation [91]. The autoxidation of hemoglobin is enhanced under hypoxic conditions in the microcirculation, especially during the formation of unstable dimers. Moreover, both forms can further react with hydrogen peroxide leading to heme degradation with the release of free iron [92]. Usually, these forms are present in plasma if hemoglobin is not bound to haptoglobin (Hp) and/or hemopexin (Hpx, Hx). It has been shown that Hb binding to Hp limits not only the reactivity of ferryl iron but also the ferryl radical form of Hb. Thus, heptaglobin plays a protective role against oxidative damage induced by Hb in plasma, not preventing the reactivity of heme oxidants, but limiting the harmful effects of products formed as a result of protein degradation [93]. In turn, hemopexin binds to free heme, which is formed during Hb degradation. Hemopexin is therefore a glycoprotein that protects against oxidative damage initiated by heme in the vascular system, especially during intravascular hemolysis [94]. Heme is released during autoxidation of Hb to MetHb and is an important source of other ROS that contribute to oxidative stress formed in plasma. Increased oxidative stress occurs when lower molecular weight Hb dimers enter the cell and tissue [92]. In addition, heme and oxyferryl exhibit proinflammatory effects by increasing oxidative stress. This situation occurs in diseases such as atherosclerosis, renal failure, sickle cell disease, and malaria. The proinflammatory effects of oxyferryl and heme lead to pathologies such as atherosclerosis, renal failure, and anemia. The effects of extracellular hemoglobin in hemolytic anemia are particularly dangerous. In such cases, blood transfusions are performed, and what is particularly important, they should not cause an increase in the concentration of extracellular hemoglobin [92].

**Figure 2:**
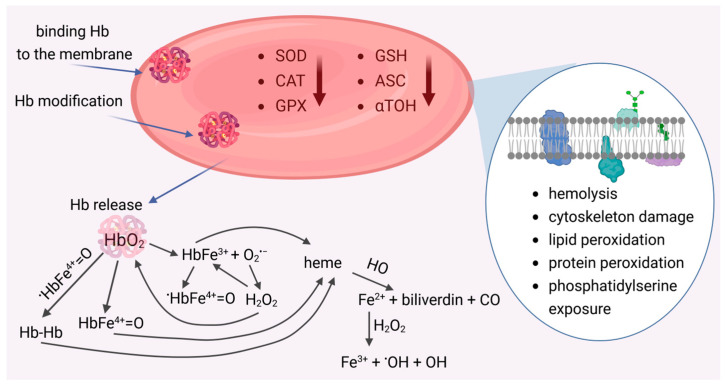
Damaged red blood cells and its pro-oxidant effects. Reduced activity of antioxidant enzymes (SOD, CAT, GPx) and low-molecular-weight antioxidants (GSH, Asc,α-TOH) promotes the release of reactive oxygen species (ROS). ROS generation occurs as a result of RBCs hemolysis and the release of hemoglobin, which, as a result of autoxidation, forms methemoglobin (HbFe^3+^) and generates superoxide anion (O_2_^•−^). Spontaneous or catalyzed dismutation of O_2_^•−^ leads to hydrogen peroxide (H_2_O_2_), which reacts with HbO_2_ to form the ferryl form HbFe^4+^=O and with MetHb the radical ferryl form (^•^HbFe^4+^=O). The HbFe^3+^ and Fe^4+^=O forms, as well as Hb dimers, can release heme. As a result of the action of heme oxygenase (HO), iron is released, which, in reaction with hydrogen peroxide, generates a radical hydroxyl (^•^OH).

Red blood cells (RBCs) are exceptionally sensitive to oxidative damage due to their functions. On the one hand, they are exposed to high oxygen concentrations in the lungs, and on the other, the hemoglobin present within the cell undergoes autoxidation, generating superoxide anion. In normal red blood cells, approximately 3% of Hb can be converted to MetHb daily [1]. However, within the cell, NADPH-dependent hemoglobin reductase occurs, which reduces MetHb to HbO_2_, preventing damage to biological material. Therefore, within the erythrocyte, a delicate balance exists between the generation of ROS and their removal by antioxidant enzymes and low-molecular-weight antioxidants. Disturbance of this balance leads to oxidative damage to metabolic functions in the intracellular environment and disruption of membrane integrity, including the cytoskeleton and lipid bilayer. Abundant evidence demonstrates the involvement of oxidative stress in red blood cell dysfunction, both in the physiological aging process and in pathologies such as diabetes, inflammation, and hemolytic disorders [95]. Under these conditions, hemoglobin leaks from RBCs, which, deprived of their antioxidant shield, become significantly more sensitive to oxidation. This leads to the formation of various reactive forms with high oxidative potential, which causes oxidative damage to molecules, macromolecules, and blood cells (including RBCs) as well as vascular cells (Figure 2). Disintegration of cell membranes and disruption of metabolic processes lead to premature removal of RBCs from the bloodstream.

### 1.6. Prooxidant Action of Heme

**Figure 3:**
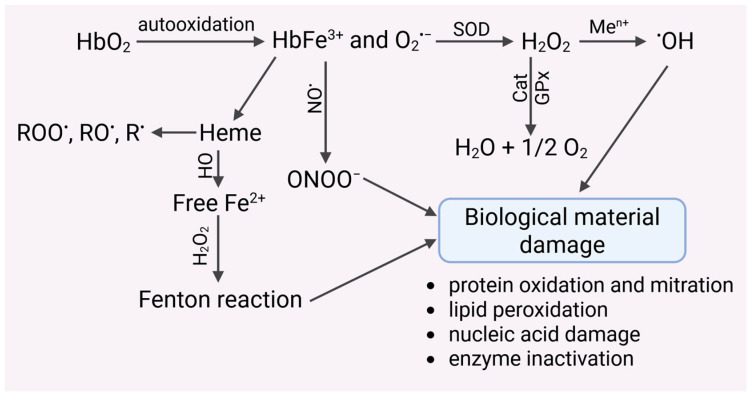
Autoxidation of hemoglobin leads to the generation of the O_2_^•−^ radical, which may undergo spontaneous or catalyzed dismutation to harmful hydrogen peroxide. H_2_O_2_ is removed by catalase, glutathione peroxidase and peroxyredoxin-2. Undecomposed hydrogen peroxide can undergo transition metal-catalyzed (Me^n+^) reduction to a highly reactive hydroxyl radical, which damages biological materials. This radical can also be generated by hemoglobin, which undergoes autoxidation to methemoglobin. MetHb is decomposed to heme, which releases iron(II) ions, catalyzing the Fenton and/or Haber–Weiss reactions. Iron chelators inhibited the formation of hydroxyl radicals.

The heme structure contains a central iron(II) ion, which forms a coordination complex in the porphyrin ring. The porphyrin ring consists of four pyrrole rings connected by methenyl bridges. The iron ion is centrally bound by nitrogen atoms present in the pyrrole rings. Furthermore, various side chains are attached to the porphyrin, including vinyl residues, histidine residues, methyl groups, and a hydroxyethylfarnesyl group (sesquiterpenoid). This coordination complex plays a key role in oxygen binding in hemoglobin. Heme has been shown to have pro-oxidant properties (Figure 3).

Autoxidation of HbO_2_ to MetHb promotes the generation of hydroxyl radicals via the Fenton reaction [96]. The presence of iron after heme decomposition can catalyze Fenton reactions, but it can also react with organic hydroperoxides to form alkoxyl (RO^•^) and peroxide (ROO^•^) radicals, and even alkyl (R^•^) radicals [97,98] (Figure 3). These radicals can initiate damage to biological material, including proteins, lipids, nucleic acids, and others. In addition, heme has a proinflammatory effect. It has been shown that sterile intra- or extravascular hemolysis led to inflammation, which is regulated by Hx and heme oxygenase-1 (HO-1) [99]. Hemin has been shown to enhance neutrophil recruitment in vivo. In turn, exposure of neutrophils to hemin led to the expression of the chemokine interleukin-8, suggesting a possible molecular mechanism involved in the induction of chemotaxis in vivo. Furthermore, hemin initiated an oxygenic burst in human neutrophils, and the amount of ROS produced was dependent on the concentration of hemin added to the cells [100]. Together with the release of heme, there is the expression of heme oxygenases (HO), which break down heme into biliverdin and carbon monoxide and release iron (Figure 3). Depending on the redox state of the cell, heme oxygenases can have antioxidant or prooxidant effects. At the same time, heme oxygenases stimulate the biosynthesis of ferritin, which binds iron [101]. Hb, which is a biological substrate for HO in microsomes, led to lipid peroxidation. This process was inhibited by HO inhibitors and iron chelators. These results suggest an iron-dependent and HO-driven mechanism. Similar studies were performed in smooth muscle cells from the rat pulmonary artery. An HO inhibitor was shown to inhibit the increase in intracellular iron following Hb treatment of cells. Furthermore, the introduction of bilirubin did not prevent the pro-oxidant effect of iron in either microsomal or liposomal systems [102]. Heme can catalyze the generation of ROS by activating enzymes such as NADPH oxidase [103]. Heme initiated VSMC migration and proliferation dependent on NADPH oxidase-derived ROS. In addition to activating redox-sensitive signaling pathways associated with proliferation, heme induced the expression of heme oxygenase vascular smooth muscle cells (VSMC). The use of HO inhibitors enhanced heme-induced proliferation and simultaneously increased ROS generation. Such results were not observed in the presence of heme metabolites, i.e., carbon monoxide and biliverdin [103].

Endogenous DAMPs (Damage-associated molecular patterns) formed as a result of cell and tissue damage can induce and/or modify innate immune responses. Hemoglobin and heme released as a result of RBC hemolysis create forms in which iron occurs in different oxidation states; these molecules can also act as DAMPs. Heme is one of the best-known DAMP molecules that interact with both immune and nonimmune cells. Heme is a chemoattractant that activates the complement system, modifies host defense mechanisms, and initiates innate immune memory [104].

### 1.7. Red Blood Cells in Inflammation

Oxidative stress, caused by an imbalance between ROS production and their scavenging, can lead to inflammation. Oxidative stress and inflammation are associated with a feedback loop, as one can induce the other and vice versa. Furthermore, both processes are involved in many pathological conditions. Oxidative stress activates numerous transcription factors, leading to the expression of certain genes involved in inflammatory pathways. In turn, inflammation initiated by oxidative stress is associated with many chronic diseases [105,106].

**Figure 4:**
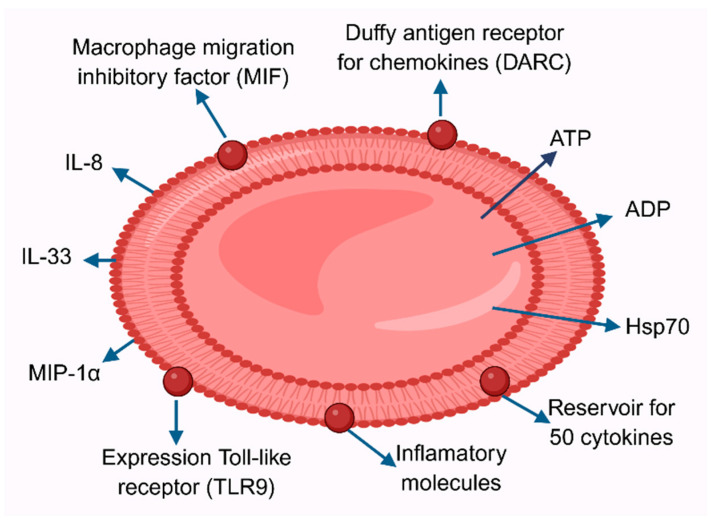
Red blood cell in inflammation. RBCs are reservoirs of approximately 50 cytokines. Furthermore, RBCs possess the Duffy receptor for chemokines (DARC). Macrophage migration inhibitory factor and TLR9 are expressed on the RBC surface. Inflammation also leads to the release of ATP and ADP, as well as heat shock proteins Hsp70, interleukins IL-8, IL-12, IL-33, and MCP-1 and MIP-1α. These factors may be present during RBC hemolysis in the plasma. Furthermore, the adhesive properties of RBCs to the vascular endothelium increase.

Red blood cells play an important role in inflammation, influencing immune function and initiating inflammatory responses following transfusion. Washing RBCs before transfusion has been shown to significantly reduce adverse effects, but the factors responsible for this have not been identified. At the end of the 19th century, Biernacki demonstrated that erythrocyte sedimentation rate (ESR) can be an indicator of inflammation [107]. This test was long used as a marker of inflammation and was replaced by C-reactive protein, a better marker for changes in the inflammatory microenvironment. Studies have shown the presence of 46 cytokines in RBCs lysates, and their concentration in RBCs was 12 times higher than in plasma (Figure 4). The results of these studies show that, in addition to their role in gas transport, RBCs participate in cytokine signaling. Interestingly, the proinflammatory cytokine and enzymes, macrophage migration inhibitory factor (MIF) were present in RBCs at concentrations 1000-fold higher than in plasma. Furthermore, RBCs can release factors that can initiate the secretion of proinflammatory markers from lung fibroblasts. These features indicate that RBCs may be an important component of the immune system, and are capable of signaling or receiving signals from other cell types [108]. The Duffy antigen receptor for chemokines (DARC) has been shown to bind chemokines from the CXC or CC cytokine families, including IL-8, RANTES, and MCP-1. The authors suggested that this receptor may be a reservoir of chemokines, and their release modulates inflammation [109]. Analyses of red blood cells revealed that they undergo significant changes during storage. Their morphology changes, and they become stiffer and more susceptible to hemolysis [110]. Abundant evidence indicates that RBCs undergo oxidative damage and acquire the properties of aging red blood cells [111].

Recently, RBCs from healthy donors have been shown to regulate the activity and maturation of immune cells and bind over 50 cytokines, acting as a reservoir for these molecules. A decline in this activity may be associated with disease progression. Mature RBCs can bind and release numerous chemokines, cytokines, and growth factors, and can interact with a wide variety of cells (Figure 2). Furthermore, they can partially regulate the level of inflammatory molecules in the blood through a buffer system, directly influence the activity of immune cells, and likely communicate with these cells [112]. Red blood cells transfusions can initiate inflammation, and frequent RBCs transfusions can cause adverse effects such as immunosuppression and increased morbidity and mortality [112].

Intravascular hemolysis, associated with the breakdown of red blood cells in the circulation, can occur as a result of numerous diseases, including genetic ones, but also in response to transfusions and infections, such as those caused by malaria or *Clostridium perfringens*. As a result of hemolysis, large amounts of DAMPs are released into the bloodstream. If they are not neutralized by innate protective mechanisms, they can activate numerous inflammatory pathways. The main DAMP molecule, heme, can activate converging inflammatory pathways, including Toll-like receptor signaling, neutrophil extracellular trap formation, and inflammasome formation. This indicates that DAMPs not only activate but also amplify inflammation. Other DAMPs released by RBCs during hemolysis include heat shock proteins (Hsp), such as Hsp70 but also IL-33, and ATP. Therefore, hemolysis is a major inflammatory mechanism associated with the clinical manifestations of hemolytic diseases, including pulmonary hypertension and leg ulcers. It can also lead to specific complications of sickle cell disease, including endothelial activation, vaso-occlusive processes, and tissue damage [113].

Recent studies have shown that Toll-like receptor 9 (TLR9), expressed on the surface of red blood cells and identifying nucleic acids, also plays a role in activating the innate immune system and eliminating RBCs during inflammation. It is believed that RBCs may also play an immune role by stimulating vascular dysfunction. Recent studies have demonstrated changes in the structure of the plasma membrane and metabolism of RBCs following SARS-CoV-2 infection. Furthermore, they may influence the host immune response through complement control, suggesting they have an immune function [114].

## 2. Conclusions

Red blood cells, which lack organelles, only have a few metabolic pathways, the purpose of which is to generate energy to ensure their functions. This property makes them sensitive to different stressful conditions, such as oxidative stress, hypoxia or hyperglycemia. These factors not only cause RBCs damage, which leads to changes in their biochemical and biophysical properties, and as a result have an impact on their deformability, rheological properties, but also on interactions with platelets and vascular endothelium. Further, interesting studies will allow us to learn about the mechanisms of interactions between RBCs and other cells, as well as signal transmission controlling the altered RBCs metabolism and destruction of these cells. Equally important will be to learn and understand the molecular changes related to the functions and biophysical properties of RBCs in the context of their participation in the development of microvascular pathologies, as well as the development of circulatory diseases and various vascular complications. Studies related to the use of appropriate pharmacological agents in mitigating RBCs damage are also necessary. Less altered RBCs would contribute to limiting microvascular disorders in patients with DM, which could reduce complications and mortality caused by hyperglycemia.

## Data Availability

The data presented in this study are available on request from the corresponding author.
